# Nucleo-cytoskeletal coupling controls intracellular deformation partitioning during cell stretching

**DOI:** 10.1098/rsos.250409

**Published:** 2025-07-30

**Authors:** Jerry Chen, Iris Sloan, Alexandra Bermudez, David Choi, Ming-Heng Tsai, Lihua Jin, Jimmy K Hu, Neil Lin

**Affiliations:** ^1^ University of California Los Angeles, Los Angeles, CA, USA

**Keywords:** nucleo-cytoskeletal coupling, cell stretching, image-based measurement, strain measurement, cell mechanics, systems biology

## Abstract

Cells sense and transduce mechanical forces to regulate diverse biological processes, yet the mechanical stimuli that initiate these processes remain poorly understood. In particular, how nuclear and cytoplasmic deformations respond to external forces is unclear. Here, we developed a microscopy-based technique to quantify the extensional uniaxial strains of the nucleus and cytoplasm during cell stretching, enabling direct measurement of their bulk mechanical responses. Using this approach, we identified a previously unrecognized inverse relationship between nuclear and cytoplasmic deformation in epithelial monolayers. We demonstrate that nucleo-cytoskeletal coupling, mediated by the Linker of Nucleoskeleton and Cytoskeleton (LINC) complex, regulates this anti-correlation (Pearson correlation coefficient approx. 0.3). Disrupting LINC abolished this relationship, revealing its fundamental role in intracellular deformation partitioning. Furthermore, we found that cytoplasmic deformation is directly correlated with stretch-induced nuclear shrinkage, suggesting a mechanotransduction pathway in which cytoplasmic mechanics influence nuclear responses. Lastly, multivariable analyses established that intracellular deformation can be inferred from cell morphology, providing a predictive framework for cellular mechanical behaviour. These findings refine our understanding of nucleo-cytoskeletal coupling in governing intracellular force transmission and mechanotransduction.

## 1. Introduction

Epithelial tissues experience mechanical forces during development [1,2], injury [3–5] and regeneration [6], and these mechanical signals can profoundly impact cell cycle progression [7], differentiation [8,9] and migration [10]. To understand mechanically induced intracellular events and downstream cell phenotypes, various cell stretching systems have been developed and utilized as foundational tools. For instance, uniaxial stretching has been found to reduce nuclear stiffness through histone modification in epidermal progenitor cells [11]. Biaxial stretching of mesenchymal stem cells enhances vascular regeneration through force-activated transcription factors [12]. Cyclic stretching of primary cortical neurons orients branch formation along with the cytoskeleton [13].

Despite significant advances in understanding the role of mechanotransduction in regulating cellular processes, the mechanisms governing intracellular strain distribution—particularly its effects on nuclear and cytoplasmic deformation—remain understudied. During tissue stretching, mechanical forces are transmitted through the cytoskeleton and nuclear envelope at distinct subcellular locations [14]. These forces not only induce intracellular deformations but also modulate the activities of local mechanosensors and mechanotransducers, leading to measured cellular responses [15–18]. It is important to note that intracellular mechanical properties are in fact heterogeneous, as different organelles exhibit distinct elastic properties, resulting in highly non-uniform deformations under strain [19]. However, many cell-stretching experiments assume a uniform cellular deformation. This oversimplification limits the ability to attribute mechanical stimuli to specific subcellular events, leaving open questions about how localized deformations in the cytoplasm and nucleus contribute to mechanotransduction and downstream cellular responses.

Light microscopy-based approaches have been widely employed to characterize intracellular deformation due to their non-invasive nature and sub-micrometre resolution [20]. These methods have revealed heterogeneous stiffness within the nucleus and its correlation with DNA content [21], demonstrated the mechanosensitivity of mitochondria through induced deformations [22] and identified inelastic deformations of focal adhesion complexes as a novel mechanism for amplifying force transmission [23].

Building on these advances, we developed an image-based approach to quantify the extensional uniaxial strain of both the nucleus and cytoplasm during bulk tissue stretching. By integrating a cell stretcher, live fluorescent imaging and segmentation-based deformation analysis, we measured how these subcellular compartments deform along the axis of the applied strain. Upon stretching an epithelial monolayer on an elastic membrane, we analysed the variation in nuclear and cytoplasmic elongation across different cells. This revealed a heterogeneous strain response to uniaxial stretching. Leveraging the natural endogenous intra-population mechanical variation within the monolayer, we observed a modest anti-correlation between nuclear and cytoplasmic strains, which was abolished upon disrupting the nucleo-cytoskeletal coupling, highlighting its critical role in strain partitioning. Furthermore, we found that cytoplasmic strain correlates with nucleus shrinkage in cyclically stretched cells, suggesting potential mechanotransduction pathways that relay cytoplasmic strain information to fine tune nucleus size. Lastly, we demonstrated that the extent of intracellular strain can be inferred from cell morphological features, providing a predictive framework for cellular mechanical responses.

## 2. Methods

### 2.1. Cell culture

Plasma membrane-GFP Madin-Darby Canine Kidney (MDCK) cells and dominant-negative (DN) Klarsicht, ANC-1 and Syne Homology-GFP (KASH-GFP) MDCK cells were maintained at 37°C with 5% (v / v) CO${,}_{2}$ and humidity in MEM-α (Fisher Scientific, 12561-056) supplemented with 10% (v/v) fetal bovine serum (FBS) (Fisher Scientific, 12662-029) and 1% (v/v) Penicillin-Streptomycin (Fisher Scientific, 15140-1220). Human telomerase reverse transcriptase-immortalized renal proximal tubule epithelial (RPTEC-TERT) cells were cultured at 37°C with 5% (v/v) CO${,}_{2}$ and humidity in Dulbecco's Modified Eagle Medium/Nutrient Mixture F-12 (DMEM/F12) (Fisher Scientific, 11330-032) supplemented with RPTEC growth kit (ATCC, 80320444), 10% (v/v) FBS and 1% (v/v) Penicillin-Streptomycin.

### 2.2. Stretching experiment

A 150 μm-thick polydimethylsiloxane (PDMS) membrane attached to stretcher jigs was fabricated as described in our previously published procedure [24]. Briefly, Sylgard 184 (base-curing agent ratio 10:1) was used for spin-coating at 2000 r.p.m. speed for 2 min on a glass coverslip that was pre-coated with 10% (m/v) polyvinyl alcohol at a speed of 1500 r.p.m. for 2 min. Two parallel cell stretcher jigs were then attached to the uncured PDMS coating, and the composite was cured at 150°C for 30 min. After autoclaving, the composite was treated with 12.5 μg ml^−1^ fibronectin (Sigma-Aldrich, F1141-2MG) and then incubated at 37°C for 30 min. The cells were then seeded on the fibronectin-treated PDMS membrane at a density of 100 000 cells per cm${,}^{2}$ 72 h before stretching experiments to allow cell–cell junctions to mature. We then mounted and aligned the cell monolayer–jig composite on the uniaxial stretcher above the optical window and pipetted 50 μl corresponding culture media on the cell monolayer (electronic supplementary material, figure S1a). We applied a 25% strain for single stretch or a 25% strain at 0.05 Hz for cyclic stretch by controlling the stretcher motors with our customized LabVIEW program.

### 2.3. Microscopy and image analysis

Fluorescent images were acquired using a confocal microscope (RCM1 with Nikon Eclipse Ti-E, NIS-Elements software) with 20×/0.75 NA, 60× WI/1.00 NA objectives. The imaging conditions were consistently maintained across all experiments. To identify cell and nucleus contours, Z-projected images obtained from z-stack imaging using a step size of approximately 2 μm were used. To reconstruct three-dimensional nucleus volume, a fine step size of approximately 0.2 μm was used for z-stack imaging. To measure the strains and cell morphology, the projected images at different stretching states were registered to remove offset by the registration tool in Fiji. Then, cell and nucleus segmentation were processed using Cellpose 2.0 [25] to generate the respective object masks. The masks of individual cells and nucleus were paired and labelled by the customized Macros code in Fiji. Lastly, nuclear *xx* strain ($\epsilon_{n}$), cytoplasmic *xx* strain ($\epsilon_{c}$), cellular *xx* strain ($\epsilon_{\text{cell}}$) (Δu/Δx) and the morphological features were measured using our customized Matlab code.

### 2.4. Generation of cell lines and inhibition experiments

To tag the plasma membrane with GFP, wild-type (WT) MDCK cells were transfected with pAcGFP1-Mem vector (Takara, 632509). To decouple the Linker of Nucleoskeleton and Cytoskeleton (LINC) complex by overexpressing a dominant-negative KASH (DN-KASH) protein, WT MDCK cells were co-transfected with vectors carrying GFP-KASH2 (Addgene, plasmid no. 187017), PiggyBac transposase and reverse tetracycline activator using Lipofectamine Reagents (Invitrogen, 18324012). After neomycin selection, GFP-positive clonal populations were isolated through fluorescence-activated cell sorting (FACS) sorting (BD FACS Aria H).

To inhibit myosin II and microtubules, the cells were treated with blebbistatin (Millipore Sigma, B0560) at 25 μM for 2 h and nocodazole (Millipore Sigma, M1404) at 10 nM for 24 h [26–28] before the mechanical stretching, respectively. These optimal concentrations were determined by titrating doses that preserve the drug’s primary inhibitory functions without causing other adverse effects in cells. The DN-KASH MDCK line was treated with doxycycline at 1 μg ml^−1^ to induce the expression of DN-KASH.

### 2.5. Canonical correlation analysis

Canonical correlation analysis (CCA) was performed using MATLAB’s built-in canoncorr function to investigate the relationship between cell deformation and morphology. In CCA, each dataset—deformation variables (strains) and morphological features—is transformed into canonical variates, which are linear combinations of the original variables weighted to maximize their correlation.

In our analysis, the deformation canonical variate was computed as a weighted combination of the three strain components (nuclear strain, cytoplasmic strain and whole-cell strain), while the morphology canonical variate was formed as a weighted combination of the 19 morphological features. The weights assigned to each strain and morphological feature were optimized to achieve the highest correlation between these two variates, allowing us to identify how morphology influences strain distribution.

Since CCA generates as many canonical correlation sets as the smaller number of variables in each dataset, our analysis produced three correlation sets, corresponding to the three strain components. This ensures that each strain component is separately analysed in relation to morphology, providing a structured way to examine their predictive relationships.

### 2.6. Data analysis and statistics

Statistical analyses were performed using GraphPad PRISM (v. 10). For all scatter plot-based correlation analyses, the Pearson correlation coefficient (*R*), confidence interval (CI) and *p*-value were calculated. To assess the statistical significance of the observed correlation, we performed 100 000 bootstrap resamples to estimate the empirical distribution of Pearson’s correlation coefficient. One-tailed *p*-values were computed by evaluating the proportion of resampled coefficients that were equal to or more extreme than the observed correlation, under the null hypothesis of no correlation (*r* = 0) in the opposite direction. To further assess the significance of correlations while accounting for multiple testing, we applied a False Discovery Rate (FDR) evaluation using a Monte Carlo simulation in MATLAB. Specifically, we randomly permuted the *X* and *Y* values of the dataset to disrupt any inherent associations and generated a null distribution of Pearson correlation values under the assumption of no true relationship. The FDR was then estimated by calculating the proportion of randomized correlations that were equal to or exceeded the observed Pearson’s R value. This approach controls for false positives and ensures that the reported correlations reflect statistically meaningful relationships (electronic supplementary material, table S2). To compare Pearson correlation coefficients between experimental conditions, we employed a pairwise bootstrap-based permutation test. Specifically, we resampled the data 100 000 times to generate an empirical distribution of the difference in correlation coefficients under the null hypothesis that the true difference between conditions is zero. A one-tailed permutation *p*-value was then computed as the proportion of resampled differences equal to or more extreme than the observed difference. *P*-values > 0.05 are denoted as not significant (n.s.), while *p*-values ≤ 0.05, ≤ 0.01, ≤ 0.001 and ≤ 0.0001 are represented as *, **, *** and ****, respectively.

## 3. Results

### 3.1. Measuring cellular and nuclear deformation in epithelial monolayers via live imaging

**Figure 1.**
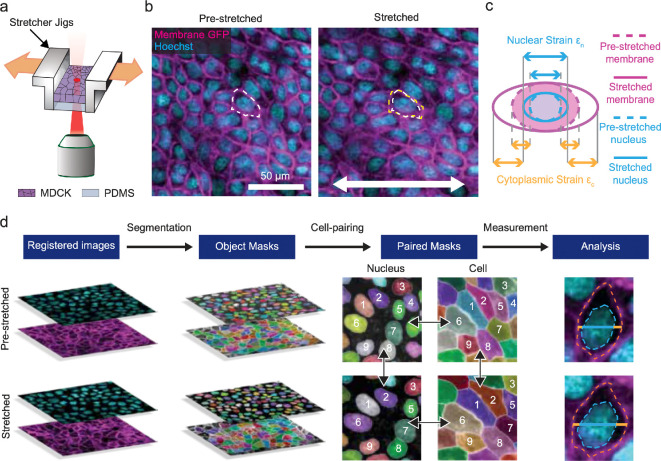
Microscopy-based uniaxial bulk strain measurements in epithelial monolayers. (a) An MDCK monolayer was cultured on a PDMS membrane. The MDCK monolayer–PDMS composite was then mounted on a custom-built cell stretcher, which applied a uniaxial tensile strain while allowing simultaneous confocal imaging. (b) Live fluorescent images of the pre-stretch (left) and post-stretch (approx. 25% strain, right) MDCK monolayer. Stretched cells exhibited nuclear and cellular morphological elongation along the axis of the applied strain. (c) Schematic illustrating the definitions of nuclear and cytoplasmic strain along the stretching direction used in this work. (d) Workflow overview outlining strain analysis pipeline. Cell and nucleus images were first registered before segmentation to obtain the respective object masks. Within the same condition (e.g. pre-stretch or post-stretch), cells were then paired with their corresponding nucleus (horizontal arrows) prior to matching pre-stretch cells to the same cell in the post-stretch image (vertical arrows). Lastly, cell and nucleus strains were quantified as illustrated in (c). MDCK, Madin-Darby Canine Kidney; PDMS, polydimethylsiloxane.

To characterize the cellular mechanical response to stretching, we developed a subcellular deformation measurement platform that integrates cell stretching, live fluorescent imaging and segmentation-based image analysis. Our custom-designed cell stretcher, featuring dual motors on opposite sides, applies symmetric uniaxial deformation with an encoder-based closed-loop control over the stretching rate and amplitude (electronic supplementary material, figure S1a) [24,29]. For deformation measurement, a confluent monolayer of membrane-GFP Madin-Darby canine kidney (MDCK) cells on a PDMS membrane was stained with Hoechst to label nuclei 30 min before stretching. Hoechst is suitable for our imaging application, as it binds to DNA without overtly affecting cell viability, allowing precise reporting of nuclear morphology and mechanics [30–32]. Beneath the mounted sample, we engineered an imaging window for live fluorescent imaging during stretching (figure 1a). By characterizing the tissue deformation using the acquired tissue images, we were able to demonstrate a high consistency with the applied strain, verifying the platform’s ability to precisely control deformation (electronic supplementary material, figure S1b).

We next quantified the deformation of the nucleus and cytoplasm, two key intracellular mechanical responses that regulate distinct mechanically induced molecular events [15–18]. Capturing fluorescent images of membrane-GFP and Hoechst allowed visualization of cell and nuclear shape changes due to uniaxial extension along the *x*-axis (figure 1b). We focused on the dominant xx strain component, defining nuclear xx strain, $\epsilon_{n}$, as the ratio of nucleus length change to its initial length, and cytoplasmic xx strain, $\epsilon_{c}$, as the ratio of cytoplasm length change to initial length and cellular xx strain, $\epsilon_{\text{cell}}$, as the ratio of total cell length change to its initial length (figure 1c). Nuclear, cytoplasmic and cellular lengths were all measured along the stretching direction, centred at the nucleus centroid. Here, we measured the strain along the extension axis mid-line, since the applied strain is relatively small, and both the cell and nucleus undergo affine deformation. We summarize the overall deformation measurement workflow in figure 1d. The pipeline quantifies the three strains from registered pre-stretch and post-stretch images through object segmentation and cell-pairing for nuclei and cells, enabling high-throughput analysis at the single-cell level.

### 3.2. Inverse relationship between nuclear and cytoplasmic extensions

**Figure 2.**
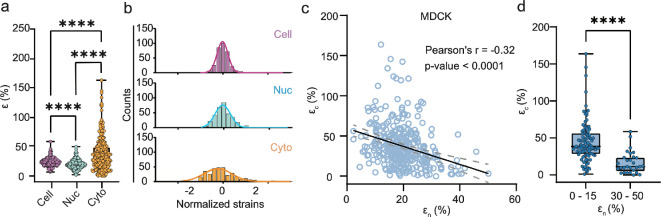
Anti-correlation between nuclear strain and cytoplasmic strain. (a) Cellular, nuclear and cytoplasmic strains for MDCK cells stretched 24%. The mean cellular strain approximately 24% was consistent with the applied tensile strain, while the mean nucleus and cytoplasmic strains were, respectively, two times lower and higher than the applied strain. *n* > 300 cells. (b) Normalized histograms for cellular, nuclear and cytoplasmic strains exhibited unimodal distributions with varying degrees of heterogeneity. Each strain was normalized to its mean. Solid lines denote a Gaussian fit for each histogram. (c) Scatter plot of nuclear strain versus cytoplasmic strain shown in (a) and (b) indicated an anti-correlation between the two strains. Solid line and dotted lines denote the best fit and 95% confidence interval, respectively. (d) Anti-correlation between the nuclear and cytoplasmic strains was confirmed by the statistically significant difference in cytoplasmic strain between the low (0–15%) and high (30–50%) nuclear strain groups. MDCK, Madin-Darby Canine Kidney.

To investigate the relationship between cytoplasmic and nuclear deformation in response to tensile stretch, we plotted the cellular, nuclear and cytoplasmic xx strains from 300 cells (figure 2a). The mean cellular strain was approximately 24%, consistent with the applied 25% strain. The mean nuclear strain was roughly half that of the mean cellular strain, while the mean cytoplasmic strain was approximately twice the mean cellular strain, consistent with previous findings showing that the nucleus is stiffer than the cytoplasm [33,34]. Normalized histograms of cellular, nuclear and cytoplasmic strains showed broad unimodal distributions for all three components (figure 2b).

We further analysed the distribution of these intracellular deformations by plotting cytoplasmic strain versus nuclear strain for all cells (figure 2c). This scatter plot revealed a moderate anti-correlation, with a Pearson’s correlation coefficient of −0.32 (*p*‐value < 0.0001) (figure 2c and electronic supplementary material, table S2). Given the modest strength of the observed correlation, it is essential to perform additional statistical analyses to rigorously validate its significance. Monte Carlo simulation confirmed a robust FDR approximately 0 for this anti-correlation (electronic supplementary material, figure S2). Here, we employ the Pearson correlation coefficient as an experimental read-out to quantify the association between the two strain components. This statistics-based read-out is commonly utilized in experimental systems biology, where results can be rigorously validated through comprehensive statistical analyses. In the later sections of this study, we will integrate this measurement with inhibition perturbations to elucidate the functional roles of specific intracellular components.

Our observed inverse relationship indicates that cells with large nuclear strains tend to exhibit smaller cytoplasmic strains and vice versa. The statistical significance of this finding was confirmed by comparing cytoplasmic strains between cells with small (0–15%) and large (30–50%) nuclear strains, resulting in a *p*‐value < 0.001 (figure 2d). This anti-correlation was also observed in an immortalized proximal tubule epithelial cell line (RPTEC-TERT), suggesting the phenomenon is not unique to MDCK cells (electronic supplementary material, figure S3).

These results indicate that the distribution of nuclear and cytoplasmic deformations is likely controlled by the mechanical coupling between the nucleus and the cytoplasm, as suggested by previous studies [35–37]. To investigate the underlying interdependence between cytoplasmic and nuclear strains, we employed a common one-dimensional spring model, conceptualizing the nucleus and cytoplasm as two springs connected in series (electronic supplementary material, figure S4a). In this model, the total strain corresponding to cell deformation is the sum of the strains of the cytoplasm and nucleus, weighted by their respective original lengths. To identify the fundamental mechanism, we excluded other cellular mechanical properties such as viscoelasticity, plasticity, Poisson’s ratio and mechanical remodelling during stretching [38–40]. We hypothesized that these higher order mechanical properties are not essential for determining the partitioning of strain between the nucleus and cytoplasm. First, we considered an extreme case where all cells exhibited identical strains ($\sigma_{\epsilon }=0$) and generated 2000 random samples, assuming equal lengths for the nucleus and cytoplasm (electronic supplementary material, figure S4b). As expected, this yielded a perfect $\epsilon_{c}-\epsilon_{n}$ anti-correlation (electronic supplementary material, figure S4c, green line) since $\epsilon_{c}$ and $\epsilon_{n}$ sum to a constant under these conditions. Next, to reflect cellular mechanical heterogeneity, we modelled strains following a Gaussian distribution with a standard deviation of $\sigma_{\epsilon_{\text{cell}}}=15\%$, finding that the anti-correlation weakened but persisted (electronic supplementary material, figure S4b,c, yellow dots). As shown in electronic supplementary material, figure S4d, we further examined strain distribution by varying the nucleus-to-cytoplasm strain ratio (i.e. stiffness ratio). A uniform ratio resulted in a weak anti-correlation (electronic supplementary material, figure S4e, yellow dots), while a broad strain ratio distribution produced a robust anti-correlation (electronic supplementary material, figure S4e, green dots). In summary, our toy model suggests three essential requirements for the $\epsilon_{c}-\epsilon_{n}$ anti-correlation: (i) a mechanical coupling between the nucleus and cytoplasm, (ii) a unimodal distribution of cellular stiffness and (iii) intracellular mechanical heterogeneity.

To gain deeper physical insights into the simulation results, we analytically derived the Pearson correlation coefficient r for the correlation between the cytoplasmic strain $\epsilon_{c}$ and the nuclear strain $\epsilon_{n}$. The Pearson correlation coefficient is defined as $r\equiv \text{Cov}(\epsilon_{c},\epsilon_{n})/(\sigma_{\epsilon_{c}}\sigma_{\epsilon_{n}})$*,* where $\sigma_{\epsilon_{c}}$ and $\sigma_{\epsilon_{n}}$ denote the standard deviations of $\epsilon_{c}$ and $\epsilon_{n}$, respectively and $\text{Cov}(\epsilon_{c},\epsilon_{n})$ is the covariance between these variables. By considering the relationship between the total cell strain $\epsilon_{\text{cell}}$, cytoplasmic strain $\epsilon_{c}$ and nuclear strain $\epsilon_{n}$, given as $\epsilon_{\text{cell}}=\epsilon_{c}+\epsilon_{n}$, the covariance can be expressed in terms of the variances $\text{Cov}(\epsilon_{c},\epsilon_{n})=\frac{1}{2}(\sigma_{\epsilon_{\text{cell}}}^{2}-\sigma_{\epsilon_{c}}^{2}-\sigma_{\epsilon_{n}}^{2})$. Substituting this expression into the definition of r, we obtain $r=\left(\sigma_{\epsilon_{\text{cell}}}^{2}-\sigma_{\epsilon_{c}}^{2}-\sigma_{\epsilon_{n}}^{2})/(\sigma_{\epsilon_{c}}\sigma_{\epsilon_{n}}\right)$. In the special case where the total cell strain is constant, i.e. $\sigma_{\epsilon_{\text{cell}}}=0$, and the nucleus and cytoplasm have equal original lengths, the variances simplify to $\sigma_{\epsilon_{c}}=\sigma_{\epsilon_{n}}$. Substituting these values, the Pearson correlation coefficient reduces to r=−1, indicating a perfect anti-correlation between the cytoplasmic and nuclear strains. This result is consistent with the simulation findings in electronic supplementary material, figure S4b,c.

Integrating the simulation results as shown in electronic supplementary material, figure S4b–e, the closed form of Pearson’s r and our experimental observations of $\epsilon_{c}$-$\epsilon_{n}$ anti-correlation reveal two key implications. First, the degree of anti-correlation between cytoplasmic and nuclear strains depends directly on the variability in mechanical deformation of the cell, cytoplasm and nucleus under stretching. Specifically, the anti-correlation becomes more pronounced as the cell’s strain response becomes more uniform, and the distributions of cytoplasmic and nuclear strains broaden. More importantly, the presence of anti-correlation indicates that $\epsilon_{c}$ and $\epsilon_{n}$ are not independent variables. Within the framework of a one-dimensional spring model, the interdependence between $\epsilon_{c}$ and $\epsilon_{n}$ arises directly from the mechanical coupling between cytoplasm and nucleus (electronic supplementary material, figure S4a). In the absence of such coupling, the stiffness heterogeneity between the cytoplasm and nucleus alone would not result in any interdependence between $\epsilon_{c}$ and $\epsilon_{n}$.

### 3.3. Nucleo-cytoskeletal coupling regulates nuclear and cytoplasmic extension in stretched monolayers

**Figure 3.**
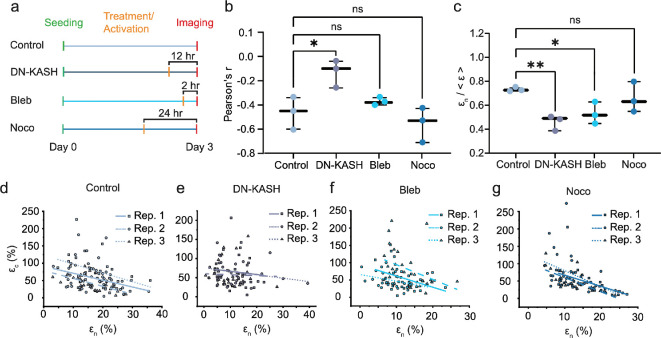
Nucleo-cytoskeletal coupling is required for intracellular strain distribution control. (a) Experiment overview investigating the role of cytoplasmic force propagation in the anti-correlation between nuclear and cytoplasmic strain. A dominant-negative KASH GFP reporter line (DN-KASH), Blebbistatin (Bleb) and nocodazole (Noco) were used for LINC complex disruption, Myosin-II inhibition and microtubule polymerization inhibition, respectively. (b) The physical decoupling of the nucleoskeleton to the cytoskeleton reduces the magnitude of the anti-correlation between nuclear and cytoplasmic strain, while the relaxation of the cytoskeleton does not play a role in generating the observed anti-correlation. (c) Disruption of LINC complex and relaxation of actomyosin tension reduce the normalized nuclear strain, while microtubule polymerization inhibition plays a secondary role in regulating the nuclear strain. Nuclear strains were normalized to the global tensile strain of each condition. *n* = 3. (d–g) Scatter plots illustrating the anti-correlation between cytoplasmic strain and nuclear strain for (d) Control (e) DN-KASH (f) Bleb (g) Noco groups. Solid lines represent the best fit line. The number of data points for each biological replicate shown in Figs. 3b and 3c is as follows: 30, 30 and 30 cells for the control; 32, 29 and 32 cells for DN-KASH; 30, 30 and 31 cells for Bleb; and 30, 32 and 30 cells for Noco. LINC, Linker of Nucleoskeleton and Cytoskeleton.

Our theoretical analysis suggests that the strain partitioning between the nucleus and cytoplasm may stem from their mechanical coupling and intrinsic mechanical heterogeneity, such as variations in organelle stiffness. In this study, we focus on mechanical coupling, leveraging available tools to perturb the nucleo-cytoskeletal linkage, which enables direct investigation of this potential mechanism. To investigate this strain-regulation mechanism, we systematically perturbed three key components involved in intracellular force transmission: the LINC complex, actomyosin and microtubules (figure 3a). First, we disrupted nucleo-cytoskeletal coupling by overexpressing a dominant-negative KASH-GFP (DN-KASH) construct [41–43], which blocks the binding of endogenous SUN to Nesprin proteins in the LINC complex that is required to physically connect the nuclear interior to the cytoskeleton. Analysing the nucleus-cytoplasm strain correlation, we found that the anti-correlation was significantly attenuated in the DN-KASH group, indicated by its near-zero Pearson correlation coefficient (figure 3b). This finding suggests that nucleo-cytoskeletal coupling is required for controlling the intracellular strain distribution, consistent with previous findings that the LINC complex plays a crucial role in force transmission to the nucleus and governs nuclear mechanosensing [36,44,45].

To test if actomyosin tension and microtubules are required for controlling nuclear and cytoplasmic deformation, we inhibited actomyosin and microtubules. We weakened actin tension by administering Blebbistatin to block non-muscle myosin II in an actin-detached state and mitigated microtubule formation using Nocodazole to inhibit microtubule polymerization [46,47]. These two experiments primarily altered cytoskeletal force transmission, leaving nucleo-cytoskeletal coupling intact. Our correlation analysis found that the anti-correlation was undisrupted in the Bleb and Noco samples (figure 3b), suggesting that actomyosin tension and microtubule assembly are not required for intracellular strain correlation [20,48–50]. Scatter plots for all conditions are shown in figure 3d. Representative images of the four conditions in electronic supplementary material, figure S5 illustrate the intact morphologies and deformation response to stretch. We did not observe other adverse effects in our pharmacological inhibition experiment (electronic supplementary material, figure S6a) [28].

Lastly, we examined how these inhibitions affected overall force transmission to the nucleus by measuring mean normalized nuclear strain. Our measurements show that both Blebbistatin and DN-KASH significantly reduced nuclear strain compared with control, consistent with previous studies [43]. In contrast, Nocodazole showed no significant effect (figure 3c). The effect of Blebbistatin was further validated by morphological and atomic force microscopy measurements, demonstrating that Blebbistatin reduced cell modulus and altered cell morphology (electronic supplementary material, figure S6a,b). We also note that while DN-KASH has been shown to upregulate RhoA activity, which can lead to increased actomyosin contractility [51], the unchanged Pearson correlation coefficient in our Blebbistatin result suggests that actomyosin contractility does not have a significant influence on the observed anti-correlation. Lastly, while Nocodazole has been shown to induce actin contractility through GEF-H1 activation [52], our Blebbistatin results suggest that even if actin contractility were enhanced, it is unlikely to counteract the effects of microtubule inhibition. However, to further elucidate this interaction, future studies would benefit from investigating the combined effects of Blebbistatin and Nocodazole treatments, as well as directly measuring GEF-H1 activity.

### 3.4. Differential association of nuclear and cytoplasmic strains with nuclear size change

**Figure 4.**
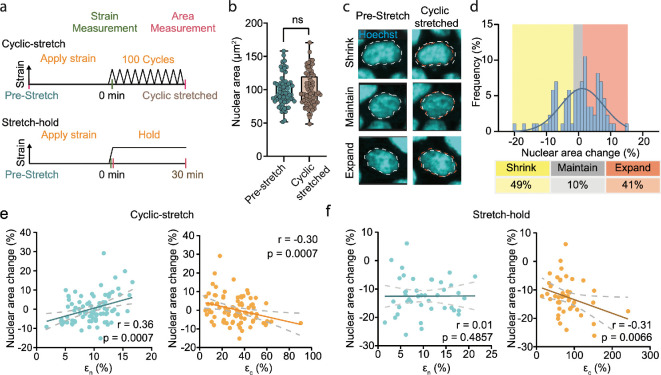
Stretch-induced nuclear size change is regulated by intracellular strain distribution. (a) Experiment overview of two different stretching profiles applied to MDCK cells. In one experiment, the initial nuclear area was measured before applying 25% strain to the monolayer and then measuring the resulting strain. Following this, the monolayer was cyclically stretched 100 times at a frequency of 0.05 Hz prior to measuring the nuclear area. The total duration of cyclic stretching is approximately 33 min. In a separate experiment, 25% strain was applied to the monolayer and the resulting strain and nuclear area was measured. The sample was then held in the stretched state for 30 min, and the nuclear area was remeasured. (b) Nuclear area change in response to cyclic stretching shows no significant difference. (c) Fluorescent images of nuclei before and after cyclic stretching. Cyclic stretching induces a heterogeneous response in nuclear size change as shrinking, no change and expansion we observed. (d) Histogram illustrating nuclear size response to cyclic stretching. Gaussian fit overlaid. (e) Scatter plots of nuclear area change versus nuclear strain $\epsilon_{n}$ (left) and nuclear area change versus cytoplasmic strain $\epsilon_{c}$ (right), respectively, demonstrate a positive and negative correlation when cyclically stretched. (f) Scatter plots of nuclear area change versus nuclear strain $\epsilon_{n}$ (left) and nuclear area change versus cytoplasmic strain $\epsilon_{c}$ (right), respectively. MDCK, Madin-Darby Canine Kidney

In this section, we investigated the biological significance of the strain partitioning between the nucleus and cytoplasm by characterizing stretch-induced nuclear mechanoresponse. Given that nuclear size change is a prominent cellular phenotype in response to mechanical stretch [53–55], we aimed to understand how intracellular strain distribution affects nuclear size change, where force-activated molecular events can vary across subcellular compartments. Following established protocols [31,56], we performed cyclic stretching to sustainedly activate mechanotransductions by recurrently deforming an epithelial monolayer (figure 4a). In this experiment, we first characterized nuclear and cytoplasmic strains across one field of view during the initial stretch cycle. We then applied 100 stretch cycles at a frequency of 0.05 Hz over approximately 33 min, followed by the acquisition of fluorescent images for nuclear size measurements. The stretching duration was limited to 33 min to minimize substantial mechanical remodelling of the actin cytoskeleton or nucleus, which could potentially influence their deformation responses. For simplicity, we approximated the mechanical response throughout the experiment using strain measurements from the first stretching cycle. To approximate nuclear size, we used the projected area, validating this approach by observing agreement between changes in three-dimensional nuclear volume and projected area in a confocal image stack (electronic supplementary material, figure S7).

Initial analysis showed no significant difference in mean nuclear area between pre-stretch and cyclically stretched samples (figure 4b). However, individual nuclei exhibited diverse responses: some nuclei shrank (≥1% area decrease), others maintained size (<1% area change) and some expanded (≥1% area increase) in response to cyclic stretching (figure 4c). This diverse response is illustrated by the broad distribution of the area change histogram (figure 4d). The observation of both nuclear shrinkage and expansion aligns with previous findings that mechanical stretch induces various molecular responses, leading to either an increase or reduction in nuclear size [11,31,57].

To investigate how nuclear size changes may be governed by the strains, we plotted area change against nuclear strain ($\epsilon_{n}$) and cytoplasmic strain ($\epsilon_{c}$) (figure 4e). We found that nuclear area change was positively correlated with nuclear strain (Pearson’s correlation coefficient approx. 0.36) and negatively correlated with cytoplasmic strain (Pearson’s correlation coefficient approx. −0.30) (detailed statistic analysis in electronic supplementary material, table S2). These correlations indicate that intracellular strain distribution influences nuclear size change during stretching. This suggests that nuclear expansion is mainly driven by nuclear deformation, while nuclear shrinkage is primarily caused by cytoplasmic deformation. Chromatin repulsion forces have been reported to regulate nuclear size [58,59]. To understand how strain partitioning is associated with the chromatin distribution, we measured the change in coefficient of variance (CV) of Hoechst signal before and after cyclic stretch. As shown in electronic supplementary material, figure S8, cells with small nuclear strains exhibited a significant reduction in CV, indicating a potential tendency for chromatin to undergo dispersion.

We also performed a stretch-hold experiment (figure 4a) and repeated the nuclear area analysis, as stretch-hold represents a different mechanical perturbation that can elicit distinct cellular responses. The stretch-hold results (figure 4f) were qualitatively consistent with the cyclic-stretch results (figure 4e), but most nuclei exhibited area reduction in the stretch-hold experiment (electronic supplementary material, figure S9a). Representative images of nuclei in electronic supplementary material, figure S9b illustrate that the heterogeneity in nuclear area change also exists. Consequently, the correlation between nuclear area change and $\epsilon_{n}$ was low in the stretch-hold experiment. We reproduced the results for cyclic-stretch and stretch-hold experiments, as shown in electronic supplementary material, figure S9c,d, to validate the observations. Collectively, our findings demonstrate that nuclear and cytoplasmic strains play differential roles in regulating nuclear mechanoresponse. Additional discussions on potential mechanical explanations are presented in §4.

### 3.5. Inferring intracellular strain response from cell morphology

We have demonstrated that nuclear and cytoplasmic deformation is tightly regulated by nucleo-cytoskeletal coupling, which plays a critical role in nuclear mechanoresponses. Building on this, we next examined whether cell morphology could serve as a predictor of deformation distribution across a stretched monolayer. This analysis is motivated by the premise that, like strain responses, cell morphology is influenced by mechanical properties such as nuclear stiffness, cortical tension and cytoskeletal viscoelasticity [60–62]. Thus, we hypothesized that the morphological features of an unstretched cell layer contain sufficient information to infer its strain distribution upon stretching. To test this hypothesis, we applied CCA [63] to investigate the relationship between cell deformation and morphological characteristics.

**Figure 5.**
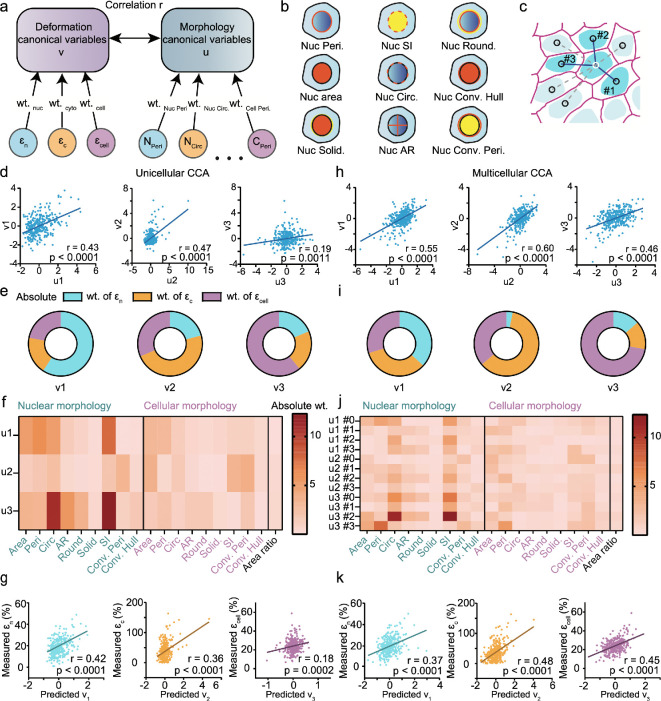
Strain distributions are correlated with cell morphology. (a) Illustration of deformation (v) and morphology (u) variables used for the canonical correlation analysis (CCA), which determines the maximum correlation between u and v by optimizing the weights (wt.) of the canonical variables. (b) Schematics of representative morphology canonical variables, including perimeter (Peri.), area, solidity (Solid.), shape index (SI), circularity (Circ.), aspect ratio (AR), roundness (Round.), convex hull (Conv. Hull) and convex perimeter (Conv. Peri.). (c) Schematic illustrating that the top three closest neighbour cells (1–3) were included in the multicellular CCA. (d) Scatter plots of canonical variates for unicellualr CCA. (e) The relative weights of nuclear strain, cytoplasmic strain and cellular strain for v1, v2 and v3. (f) Heatmap of the weights of the morphological features for u1, u2 and u3. (g) Scatter plots of measured strains versus predicted variates for unicellular CCA. (h) Scatter plots of canonical variates for multicelluar CCA, which exhibited higher correlations compared to the unicellular approach. (i) The relative weights of nuclear strain, cytoplasmic strain and cellular strain for v1, v2 and v3. (j) Heatmap of the weights of the morphological features for u1, u2 and u3, as well as the target cell (#0), closest (#1), second-closest (#2) and third-closest (#3) neighbour cells. (k) Scatter plots of measured strains versus predicted variates for multicellular CCA. CCA, Canonical Correlation Analysis.

As shown in figure 5a, CCA identified the combinations of deformation and morphological variables that are most strongly correlated, revealing how cell shape relates to strain responses. CCA was chosen over a single-variable analysis because it captures the complex, multivariate relationships between multiple morphological features and strain components rather than assessing isolated pairwise correlations. This allows for a more comprehensive understanding of how different aspects of cell and nuclear morphology collectively influence deformation.

The deformation variables included nuclear strain ($\epsilon_{n}$), cytoplasmic strain ($\epsilon_{c}$) and whole-cell strain ($\epsilon_{\text{cell}}$). The morphology variables were consisted of 19 features describing cell and nuclear shape, with representative examples shown in figure 5b and electronic supplementary material, table S1. Since strain responses are influenced by the mechanical properties of surrounding cells in a confluent monolayer, we performed an additional CCA to account for multicellular effects. In this analysis, we included the morphological features of the three nearest neighbouring cells to capture intercellular mechanical interactions (figure 5c). Each CCA result provides three correlation coefficients, corresponding to different combinations of the three strain components ($\epsilon_{n}$, $\epsilon_{c}$ and $\epsilon_{\text{cell}}$) that we aim to predict from morphological features.

In the unicellular CCA, we found that the correlation between deformation and morphology variables was strongest for the first two canonical variates (figure 5d), with nuclear or cytoplasmic strain carrying the dominant weight among the three deformation components (figure 5e). In CCA, canonical variates represent the optimal linear combinations of strain components (v-set) and morphological features (u-set) that maximize their correlation. The third variate exhibited a weaker correlation, with whole-cell strain contributing the most weight.

By examining the canonical weights of morphological features (figure 5f), we observed that nuclear morphology consistently had a stronger influence than whole-cell morphology in predicting strain responses. This suggests that nuclear shape descriptors, such as circularity (area/perimeter${,}^{2}$) and shape index (perimeter/area${,}^{1/2}$), are key predictors of nuclear and cytoplasmic deformation. To assess the predictive capability of CCA, we compared measured strain values with those inferred from the canonical variates (figure 5g). We found that nuclear strain ($\epsilon_{n}$) was best captured by the first variate (Pearson correlation coefficient approx. 0.42), while cytoplasmic strain ($\epsilon_{c}$) was most correlated with the second variate (Pearson correlation coefficient approx. 0.36). However, whole-cell strain ($\epsilon_{\text{cell}}$) was not well predicted, suggesting that additional mechanical factors, beyond those captured by the analysed morphological features, contribute to the cell’s overall deformation.

In the multicellular CCA, we observed an overall improvement in the correlation between deformation and morphology variables (figure 5h), indicating that incorporating neighbouring cells enhances predictive accuracy. Compared with the unicellular analysis, cytoplasmic strain and whole-cell strain carried greater weights in the multicellular CCA than in the unicellular CCA (figure 5i), suggesting that intercellular mechanical interactions influence these deformation components. Also, nuclear morphology remained the dominant predictor of intracellular strain, consistent with the unicellular CCA analysis (figure 5j). Including morphological features from neighboring cells improved predictive accuracy, highlighting the role of intercellular mechanical interactions in strain distribution [64].

While nuclear strain was well predicted from single-cell morphology (r approx. 0.42), cytoplasmic and whole-cell strains showed stronger correlations in the multicellular CCA (r approx. 0.48 and r approx. 0.45, respectively), compared with the unicellular model (r approx. 0.18). These results suggest that nuclear strain is largely cell-intrinsic, whereas cytoplasmic and whole-cell strains depend more on the surrounding mechanical context.

## 4. Conclusion and discussions

### 4.1. Partitioning of nuclear and cytoplasmic strains

In this study, we developed an experimental approach to quantify the extensional uniaxial strains of the nucleus and cytoplasm in stretched epithelial monolayers, enabling direct measurement of their bulk deformation. Using this method, we uncovered a previously unrecognized biomechanical role of nucleo-cytoskeletal coupling in regulating strain partitioning between these compartments. While prior studies have established that nucleo-cytoskeletal coupling transmits extracellular forces to the nucleus [36,44,45], driving nuclear remodelling [30], chromatin modification [11] and gene expression changes [65], our findings reveal its additional function in modulating the relative magnitudes of nuclear and cytoplasmic strains during uniaxial extension. Focusing on the LINC complex, we specifically disrupted the Nesprin-SUN binding and obtained results demonstrating that mechanical coupling between the nucleus and cytoskeleton, along with intracellular force balance, actively governs the observed inverse relationship between nuclear and cytoplasmic strains. These findings establish the LINC complex as a key regulator of nuclear-cytoplasmic strain partitioning.

Future investigations should explore alternative nucleo-cytoskeletal coupling pathways that may further influence nuclear and cytoplasmic strain dynamics. For instance, the perinuclear actin cap, known to constrain vertical nuclear dimensions [66,67], and the endoplasmic reticulum, which serves as a structural and mechanical link between the plasma membrane and nucleus [68], represent promising avenues for further research. These studies will help elucidate the broader mechanical network that governs strain transmission across subcellular compartments and its implications for nuclear mechanotransduction.

To fully assess factors contributing to the reduction in anti-correlation beyond nucleo-cytoskeletal decoupling, we examined the effects of both myosin II inhibition and disruption of the nucleo-cytoplasmic linkage in correlation analysis. In both cases, nuclear strain decreased, likely due to a reduction in force transmission. This decrease led to a narrowed strain ratio distribution (i.e. an altered effective stiffness ratio between the nucleus and cytoplasm), which our Monte Carlo simulations suggest could weaken the anti-correlation. The persistence of anti-correlation suggests that compensatory mechanisms, such as residual actin-LINC attachment or alternative tension pathways, may sustain force transmission, indicating that inhibiting individual cytoskeletal components alone may be insufficient to fully disrupt intracellular mechanical balance. Also, because nucleo-cytoskeletal decoupling inherently lowers nuclear strain and alters strain ratio distributions, disentangling its specific effects from other contributing factors requires a more integrated approach. A combination of theoretical modelling and systematic experimental perturbations will be crucial for precisely deciphering the role of nucleo-cytoskeletal coupling, enabling controlled variations in force transmission and direct measurements of nuclear and cytoplasmic strain responses [49,69]. This approach will provide deeper insights into the mechanisms governing strain partitioning and mechanotransduction within the cell.

### 4.2. Regulation of nuclear size changes in response to mechanical strains

Our results reveal distinct associations between nuclear and cytoplasmic strains and stretch-induced nuclear size changes, supporting the concept of nuclear mechanosensing as a protective response against mechanical stress [31,55]. Specifically, nuclear expansion correlates with nuclear strain, while nuclear shrinkage is linked to cytoplasmic deformation. These opposing trends may reflect distinct mechanotransduction pathways involving nuclear and cytoplasmic compartments [15,59,70,71]. Future studies integrating strain measurements with perturbation experiments and high-resolution imaging could clarify the molecular mechanisms involved [72–74].

### 4.3. Association between cell morphology and strain response

We further demonstrated that cell morphological features can be used to infer the intracellular strain response. This finding aligns with recent experiments illustrating a strong connection between cell morphology and traction forces [75], as well as mechanical properties [24] and biochemical responses to stimuli [76]. Our results also suggest that cell–cell mechanical connections regulate individual cell deformation in a stretched confluent epithelium, as evidenced by the enhanced prediction accuracy in multicellular CCA. This observation is consistent with previous measurements of supracellular strain [24], emphasizing the significance of multicellular mechanical coupling.

### 4.4. Future work

While this study focused on strain measurements, future work could incorporate stress- or modulus-based readouts to complement deformation analysis, particularly in stiff or minimally deforming cells [77]. Because intracellular mechanosensors operate largely independently of surrounding stiffness [15,16,70], strain remains a relevant proxy for mechanotransduction activity. Integrating molecular strain sensors, such as Förster resonance energy transfer (FRET)-based probes or membrane tension reporters [43,78], could provide mechanistic insights into localized force transmission.

## Data Availability

All data necessary to reproduce the figures in this manuscript, along with the raw data for the canonical correlation analysis, are publicly available. Additionally, custom MATLAB scripts for strain and area measurements, canonical correlation analysis and false discovery rate calculations are accessible. All these files can be downloaded via the following link [79]. Supplementary material is available online [80].
